# Role of Nutrients and Foods in Attenuation of Cardiac Remodeling through Oxidative Stress Pathways

**DOI:** 10.3390/antiox11102064

**Published:** 2022-10-20

**Authors:** Taline Lazzarin, Leonardo Rufino Garcia, Danilo Martins, Diego Aparecido Rios Queiroz, Carolina Rodrigues Tonon, Paola da Silva Balin, Bertha Furlan Polegato, Sergio Alberto Rupp de Paiva, Paula Schmidt Azevedo, Marcos Minicucci, Leonardo Zornoff

**Affiliations:** 1 Internal Medicine Department, Botucatu Medical School, São Paulo State University (UNESP), Botucatu 01049-010, Brazil; 2 Surgery and Orthopedics Department, Botucatu Medical School, São Paulo State University (UNESP), Botucatu 01049-010, Brazil

**Keywords:** antioxidants, heart failure, cardiac remodeling, food

## Abstract

Cardiac remodeling is defined as a group of molecular, cellular, and interstitial changes that manifest clinically as changes in the heart’s size, mass, geometry, and function after different injuries. Importantly, remodeling is associated with increased risk of ventricular dysfunction and heart failure. Therefore, strategies to attenuate this process are critical. Reactive oxygen species and oxidative stress play critical roles in remodeling. Importantly, antioxidative dietary compounds potentially have protective properties against remodeling. Therefore, this review evaluates the role of nutrients and food as modulators of cardiac remodeling.

## 1. Introduction

Cardiac remodeling (CR) is defined as a group of molecular, cellular, and interstitial changes that manifest clinically as changes in the heart’s size, mass, geometry, and function after injury. Importantly, remodeling is associated with poor outcomes, mainly because of an increased risk of ventricular dysfunction and cardiovascular death. Therefore, strategies to attenuate this process are fundamental in different clinical scenarios [1].

Reactive oxygen species (ROS) and oxidative stress (OS) play critical roles in CR following cardiac injury. OS may result from increased production of ROS and/or dysfunction of the antioxidant defense [2]. The balance between ROS production and inactivation is known as the redox state [3].

ROS or reactive nitrogenated species (RNS) include free radicals and nonradicals with high reactivity. They are represented by singlet oxygen, superoxide anion radicals, hydroxyl radicals, and hydrogen peroxide [3,4]. RNS, such as peroxynitrite, nitric oxide, copper, iron, and sulfur species, have also been reported [5,6]. The interaction between ROS and RNS, products of OS, proteins, lipids, and nucleic acids, determine structural changes and functions responsible for multiple pathological processes, including CR.

Considering that the dyshomeostasis of nutrients can induce OS, antioxidative dietary compounds potentially have protective properties against remodeling. The application of natural antioxidants in CR has long been studied. Therefore, this review evaluates the role of nutrients as modulators of CR. Table 1 and Table 2 summarizes the studies, their respective CR benefits, and antioxidant effects of nutrients. We included only the effects with statistical significance in the original study in the table.

For ease of reading, we divided the nutrients into four sections: Diets (ketogenic diet, hypocaloric diet), Macronutrients (fats/lipids, carbohydrates, proteins), Micronutrients (vitamins, minerals), and Phytochemicals/Phytonutrients. The markers of OS described in this review included ROS; products of oxidative damage in deoxyribonucleic acid (DNA), lipids, and proteins; and evaluation of endogenous components in pro-oxidative and antioxidative systems [7].

## 2. Diets

Two diet types have benefits in CR: the hypocaloric diet (HD) and ketogenic diet (KD). Caloric restriction of the diet in obese mice attenuated cardiac hypertrophy with a reduction in LVPWT associated with smaller cardiomyocytes. In addition, the reduction of OS was demonstrated by the lower expression of antioxidant enzymes, HO-1 and NAD(P)H: quinone oxidoreductase 1 [8]. In a metabolic syndrome model, HD reduced CR, as assessed by smaller heart weight, LV end-diastolic dimension, interventricular septum thickness, LVPWT, and LV mass. This was accompanied by an improvement in diastolic dysfunction, assessed by the ratio of early-to-late ventricular filling velocities (E/A), isovolumic relaxation time, deceleration time, the time constant of isovolumic relaxation, the ratio of LVEDP to LV end-diastolic dimension, and TI. Importantly, OS was attenuated. The activity of NOX and expression of genes (p22phox, gp91phox, p47phox, and p67phox) were downregulated in HD [9].

The KD also reduced CR. In a model of aging-associated cardiac hypertrophy, KD reduced LV end-systolic diameter, CSA, and heart weight and increased fractional shortening. The antioxidant effect was shown by enhanced activities of SOD, GPx, and CAT added to the reduction in lipid and protein damage markers, MDA, and 3-nitrotyrosine, respectively [10].

## 3. Macronutrients

### 3.1. Taurine

Taurine is a nonessential amino acid that plays multiple roles in OS. It reverses the adverse effects of excessive OS via various mechanisms, including protecting cells from mitochondrial OS and reducing ROS activity [11,12]. Taurine supplementation attenuated CR, as demonstrated by an increase in LA, LV mass, LV posterior wall thickness (LVPWT), and interventricular septum thickness induced by infarction. OS was attenuated with a lower expression of nicotinamide adenine dinucleotide phosphate oxidase (NOX) genes in the taurine group [13].

### 3.2. Fatty Acids

Dietary supplementation with n-3 polyunsaturated fatty acids appears to have antioxidant properties. In rats with aortic stenosis, flax lignan concentrate and omega-3-fatty acid combination significantly improved the contractility index, restored diastolic parameters such as LVEDP, LVSP, and LV pressure rise (dP/dt) close to normality, and reduced cardiac weight. The antioxidant effect was demonstrated by the increased activity of antioxidant enzymes and decreased MDA levels [14]. Another omega-3 evaluation showed an improvement in histological appearance with O_3_ supplementation and smaller detection of apoptotic cells in a model of toxicity induced by doxorubicin. The antioxidative effects were also estimated based on enzymatic activity and MDA levels [15].

Alpha-linolenic acid (ALA) improved cardiac function, with increased LV end-diastolic volume, stroke volume, and ejection fraction, in a model of toxicity induced by doxorubicin. The histopathological damage was also attenuated and included the myofibrillar structure and cardiomyocyte necrosis. OS was attenuated by increased detoxification enzymes (SOD, GPx, and CAT) and decreased lipid peroxidation product, MDA. In addition, this study explored the signaling involved in ALA antioxidative effect and found an improvement in Nrf-2 levels [16].

Sardine oil was encapsulated in vanillic-acid-grafted chitosan, an antioxidant material. This process was used to control release behavior and protect against oxidation in rats treated with doxorubicin. The results showed a reduction in ROS generation and lower levels of caspase-3, a participant protein of apoptosis [12].

## 4. Micronutrients

### 4.1. All-Trans-Retinoic Acid

All-trans retinoic acid (ATRA) is an active metabolite of vitamin A (VA), a fat-soluble vitamin with long chains that typically allows the structure to exert ROS scavenging properties. Usually, this activity is involved in the prevention of lipid peroxidation [17]. This topic has been summarized as an evaluation of VA-related compounds [18].

Four studies evaluated ATRA supplementation. Two in vitro studies evaluated cellular viability using rat cardiomyocytes. They showed a reduction in cellular death with ATRA administration mediated by the reduction of ROS generation and the regulation of detoxifying enzyme systems such as superoxide dismutase (SOD) and heme oxygenase-1 (HO-1) [19,20]. One of the mechanisms is increased nuclear factor erythroid-2-related factor 2 (Nrf2), a transcription factor that regulates the expression of antioxidant enzymes and several important genes related to redox homeostasis.

In experimental models, a study showed that ATRA inhibited pressure overload-induced concentric cardiac hypertrophy and prevented the progressive decline in diastolic heart function [21]. In addition, ATRA mediates its activities against damage at histopathological levels [22]. Both models interfere with the antioxidant enzyme levels and OS.

### 4.2. Beta-Carotene

One study evaluated the supplementation of beta-carotene (BC), a precursor of VA [23], in rats exposed to cigarette smoke. The results showed that the supplementation attenuated echocardiographic alterations in left ventricular (LV) end-diastolic diameter (LVEDD), LV weight, and left atrium (LA) weight. Oxidative damage was assessed by measuring lipid hydroperoxide (LH) levels. In this study, BC-supplemented animals had lower LH levels than the non-BC group.

### 4.3. Folic Acid

Folic acid (FA) is a B-complex vitamin, denoted as B9. The FA antioxidant effect is mediated by multiple mechanisms, including the reduction of homocysteine and ROS levels [24]. Experimental evidence has shown that supplementation with FA is associated with a reduction in cardiomyocyte proliferation in rats exposed to monocrotaline [25]. In aging rats, FA reduced myocytes’ cross-sectional area (CSA) [26] and reduced apoptosis and fibrosis. Two studies showed attenuation in systolic dysfunction, with a better LV ejection fraction than the respective aggressive myocardial models [26,27]. The interference in OS was shown by reduced malondialdehyde (MDA) and 8-hydroxydeoxyguanosine (8-OHDG) levels or by detoxifying enzyme modulation.

### 4.4. Vitamin E

Vitamin E (VE) is a fat-soluble compound in biological membranes that promotes the main antioxidant effect by inhibiting lipid peroxidation. A streptozotocin diabetes-induced model was developed to test VE supplementation. Vitamin supplementation improved LV function measured by echocardiographic parameters, increased LV systolic pressure (LVSP), and reduced LV end-diastolic pressure (LVEDP). The measured antioxidative parameters were a decrease in oxidized glutathione levels and myocardial 8-iso-prostaglandin F2α, a member of the prostaglandin family produced from the oxidative reaction; they could be a specific and sensitive quantitative index of OS [28].

### 4.5. Combinations of Vitamins

A study evaluated the combination of beta-carotene, ascorbic acid, and alpha-tocopherol in rabbits with pacing-induced cardiomyopathy. The administration of these antioxidant vitamins improved the rate of LV pressure, attenuated the increase in the end-diastolic dimension, and increased fractional shortening in the animals [29].

Another study evaluated the administration of vitamins C and E to rabbits with myocardial infarction (MI). The combined treatment reduced LV dilation and dysfunction. The reduction in OS was assessed by elevation of the reduced glutathione/oxidized glutathione (GSH/GSSG) ratio and reduction of OS damage markers 8-OHDG (indicates DNA damage) and 4-hydroxy-2-nonenal (4-HNE), a product of lipid peroxidation [30].

### 4.6. Zinc

Zinc (Zn) is an essential mineral in the human body. The antioxidant properties of Zn are believed to result from indirect mechanisms, mostly by the catalytic and structural roles of many proteins. Up to 10% of the proteins encoded by the human genome use Zn as a cofactor, including antioxidant enzymes [31]. In the MI model [32], Zn interferes with the echocardiographic Tei index (TI), a myocardial performance index that combines time intervals related to systolic and diastolic functions, showing an attenuation in global cardiac dysfunction. Left ventricle posterior wall velocity (LVWT) increased with Zn supplementation, and it has been proposed to evaluate total LV performance [33]. In addition, Zn reduced the diastolic marker LA diameter and E/E’ ratio, suggesting improved diastolic function. In a diabetic model, the findings included a reduction in ventricular pressure and cardiac fibrosis [34]. Considering the participation of OS in these models, using Zn in MI changed the levels of catalase (CAT), GSH, and SOD. In a diabetic model, metallothionein, an antioxidant protein efficient in scavenging ROS, was decreased [34].

### 4.7. Selenium

Selenium (Se) is a trace element essential for the function of several enzymes, including selenoproteins. The most relevant selenoproteins include glutathione peroxidases (GPx) and thioredoxin reductases (TrxR). Both are examples of regulators of redox homeostasis [35]. In the CR context, an evaluation of Se dietary intake after MI showed a cardioprotective effect with significantly different levels of GPx and TrxR [36]. Here, we highlight that the dose of Se to be supplemented should be carefully determined, as the extremes had deleterious effects. Se deficiency leads to myocardial fibrosis, systolic dysfunction, and increased OS. However, Se supplementation can also be reversed with fibrosis and diastolic dysfunction, despite lower OS markers [37]. Therefore, Se deficiency and improper supplementation lead to abnormal myocardial matrix remodeling and dysfunction in the normal heart.

### 4.8. Magnesium

Magnesium (Mg) is a divalent mineral cation and a cofactor for more than 300 enzymes in humans. Hypomagnesemia has been described as an OS promoter that impairs mitochondrial function, increases ROS generation, activates proinflammatory cells with oxidant capacity, increases catecholamine levels, and activates renin–angiotensin–aldosterone [38].

In a model of hypertension induced by N(ω)-nitro-L-arginine methyl ester treatment, a nonspecific nitric oxide synthase inhibitor, Mg supplementation prevented hypertrophy secondary to hypertension and restored contractile dysfunction. The OS balance was measured by superoxide anion and hydrogen peroxide levels, which are substances evolved during OS production in cardiovascular cells. Another marker is the carbonyl level, an oxidate protein that can be generated by the action of ROS [39].

## 5. Phytochemicals/Phytonutrients

We included in this section foods that, although they contain a variety of micronutrients and phytochemicals, their beneficial actions are attributed to phytonutrients and plants.

**Figure 1:**
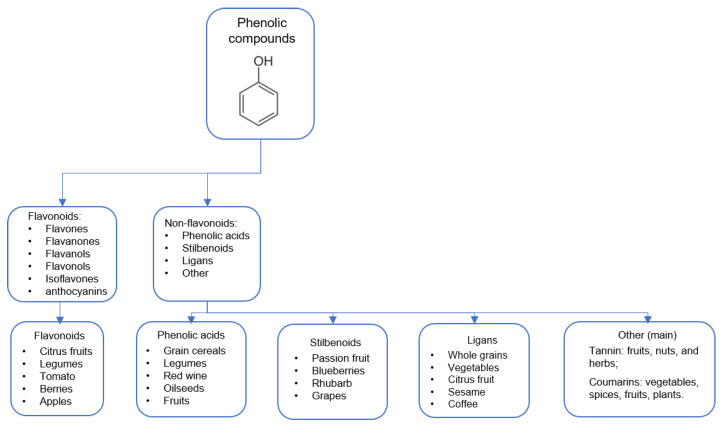
Phenolic compounds and derivates.

Using foods as antioxidant sources can be an interesting strategy because a single food can be formed by several antioxidative compounds that can attenuate OS at different points. Foods include here actuates OS by their phenolic compounds (PC). PCs are plant metabolites with a typical phenol structure (hydroxyl group on an aromatic ring) [40] usually found in fruits, vegetables, cereals, and beverages. PCs combat oxidative damage, especially due to potent free radical scavenging, decreasing protein carbonylation, and lipid peroxidation attenuation. They form a heterogeneous group with more than 8000 previously described compounds [41]. These compounds are divided into various subclasses based on their chemical structures. Below, in Figure 1, we described the main PC classes with sources [42,43,44].

Plants have been widely used in healthcare, including in preventing and managing cardiovascular diseases. Several plants have medicinal uses, but here we describe the use of edible plants with potential antioxidant effects in attenuating CR.

### 5.1. Tomato

Tomato is a red fruit that contains carotenoids, folate, PC, and vitamins C and E [19]. Its antioxidant effects include scavenging oxygen and peroxyl radicals and modulating the production of antioxidant enzymes (such as SOD, CAT, and TRx) through the activity of a common transcription factor, Nrf2. In Table 2, we synthesized studies that have also shown that in different models of cardiac injury, tomato supplementation attenuates cardiac dysfunction mediated by OS [45].

### 5.2. Spondias

Spondias is a tropical fruit rich in PC (phenolic acids and flavonoids) that effectively reduces lipid peroxidation and protein oxidation in vitro [46]. All the plant parts exhibit antioxidant effects [47]. Table 2 describes two studies using pulp and one using leaves for supplementation. The aggression induced by tobacco or infarction caused cardiac hypertrophy, which Spondias attenuated. Lipid products of OS after ingestion of this fruit were reduced in both models [48,49]. In addition, an angiotensin-converting enzyme inhibitor-like cardioprotective effect was found with Spondias compared to ramipril [50].

### 5.3. Açaí

Açaí, scientifically named *Euterpe oleracea* Mart, is a Brazilian fruit rich in PC, such as phenolic acids, flavonoids, and anthocyanins [51]. In experimental models, açaí supplementation reduced ROS generation [52]. As shown in Table 2, three studies supported the antioxidant properties of this compound. All studies found attenuation in OS with açaí supplementation. Although, only two of the three studies demonstrated evidenced improvement in CR, including a reduction in hypertrophy parameters such as heart weight, LV wall thickness, and septum wall thickness [53], and improvement of cardiac function (recovering fractional shortening) [54]. However, one study showed that açaí worsened diastolic function after ischemia–reperfusion injury. The explanation is that the injury model could cause damage by other mechanisms independent of OS suppression.

### 5.4. Jaboticaba

The small and purple fruit is rich in PCs, especially flavonoids such as anthocyanins, which are responsible for reducing cellular damage caused by OS [55]. These compounds can eliminate free radicals [56]. Infarcted rats showed improved diastolic function in echocardiograms and decreased myocardial deposition of collagen with Jaboticaba supplementation [57]. In rabbits, supplementation with the extract of jaboticaba produced cardioprotection and prevented doxorubicin-induced cardiotoxicity [55].

### 5.5. Bergamot

Bergamot is a fruit with unique flavonoid and polyphenol profiles. This profile promotes protective activities in managing atherosclerosis and metabolic disorders, especially owing to its antioxidant effect [57]. Table 2 shows a study that evaluated the role of bergamot in attenuating doxorubicin-induced cardiomyopathy by suppressing OS. Echocardiography showed a reduction in LV end-diastolic and systolic diameters associated with increased fractional shortening and ejection fraction [58].

### 5.6. Orange Juice

Orange is a citrus fruit of various varieties. Oranges are generally rich in vitamin C and flavonoids, which support antioxidant activities. We found two studies that evaluated the effect of orange juice supplementation in models of MI and doxorubicin [59,60]. One of these studies evaluated different orange juices, including Pear and Moro. Both orange juice types attenuated the structural and systolic functional changes induced by doxorubicin. However, only Moro orange juice improved diastolic function. Moro orange has become more prominent in literature because of its better antioxidant composition than Pear orange [60].

### 5.7. Raspberry

Raspberries are small and sweet fruits considered a great source of several essential micronutrients, dietary fibers, and polyphenolic components that may help decrease OS and cellular damage. We found an experimental study that evaluated the proteomics dates and reported reduced levels of proteins associated with response to OS in the raspberry group, including the NADPH dehydrogenase quinone 1, glutathione S-transferase (GST) superfamily members GST-alpha 4, and GST P1. Consistent with these findings, raspberries also downregulated proteins involved in CR [61].

### 5.8. Blueberry

Blueberry is a small fruit rich in polyphenols. Table 2 describes Louis et al.’s study, which evaluated the efficacy of five wild blueberry polyphenolic fractions (BPF) in in vitro models of norepinephrine exposure to induce OS. The previous administration of BPF prevented an increase in OS, cell death, and cardiomyocyte hypertrophy, which are associated with better contractile function [62].

### 5.9. Cranberry

Cranberry is a small fruit consumed as juice and sauce. The active constituents of cranberries include several flavonoids; therefore, their antioxidative activities were explored. Supplementation with this fruit protected against doxorubicin-induced cardiotoxicity. In fact, histological lesions such as cytoplasmic vacuole formation, interstitial edema, and fibrotic bands were significantly reduced in the supplemented rats. The antioxidant activity was assessed by regulating enzymes such as GPx and myeloperoxidase, a leukocyte-derived enzyme that catalyzes ROS formation [63].

### 5.10. Cocoa

Cocoa beans are seeds of the fruit of the Theobroma cacao tree. It is rich in flavonoids. One study tested cocoa combined with carob in rats. The motive for making this combination is that cocoa is a bean with a bitter taste, usually combined with sugar for a better taste, thus generating products with a reduced nutritional profile. However, carob is a legume that can be a good substitute for cocoa, with a more agreeable taste and improved nutritional value than the cocoa–sugar combination [64]. The cocoa–carob blend diet, which is rich in flavonoids, was evaluated alone or in combination with metformin in diabetic rats. The results showed attenuated cardiac dysfunction, hypertrophy, and fibrosis compared with the control group. The proposed mechanisms include the downregulation of NOX and modulation of the cellular antioxidant defense system, of which sirtuin-1 and Nrf2 are part [65].

### 5.11. Rosemary

Rosemary is a culinary spice that contains high levels of antioxidant compounds. Most pharmacological effects of rosemary are a consequence of the antioxidant activities of its main chemical constituents. Table 2 describes the results of rosemary supplementation at different doses in infarcted rats. The smallest dose of rosemary supplementation resulted in a more significant improvement in heart function, which was associated with decreased cardiac hypertrophy and hydroperoxide levels [66]. This fact draws attention to the dose-response importance, as described for the supplementation of several compounds.

### 5.12. Camellia sinensis

Camellia sinensis leaf infusions produce green tea (GT). GT is popular and rich in polyphenols. Catechins are the main PCs found in GT. They can interact with biological matter through hydrogen bonding or electron and hydrogen transfer. These processes promote antioxidant effects, such as scavenging ROS and RNS, inhibiting pro-oxidant enzymes, and inducing antioxidant enzymes [67]. This is the only nutritional approach beneficial in clinical models. As shown in Table 2, Calo et al.’s study showed a clinical trial of GT administration in dialytic patients. Three markers demonstrated the OS reduction: p22phox reduction, a subunit of NOX, a protein responsible for ROS generation; increased HO-1, an HO isoform that protects against OS; and the state of phosphorylation of extracellular signal-regulated kinases 1/2, and OS effector protein for cardiovascular remodeling [68]. Experimental studies in different models reinforce the beneficial effects of GT in mitigating OS and CR.

### 5.13. Moringa

Moringa is an edible plant used in food and medicine, especially in Asian countries. It is rich in natural antioxidant compounds, such as vitamins C, E, and A, phenolics, and flavonoids, which confer free radical scavenging activity [69]. As shown in Table 2, two aggressive myocardial models were evaluated. Moringa supplementation showed a reduction of gp91phox, a subunit of NOX, a protein responsible for ROS generation [70]. When evaluating different doses of moringa, although benefits are found in all doses, an intermediary dose proved more beneficial [71]. However, another study, which is not included in Table 2 because it did not evaluate oxidative regulation, showed echocardiographic benefits with better diastolic function, assessed by the reduction of isovolumetric relaxation time and deceleration time of the E wave, increase in ejection volume, and cardiac output with moringa supplementation [72].

### 5.14. Citronella

Citronella, scientifically known as Cymbopogon spp., has antioxidative effects. Culinary uses include tea preparation and flavoring of soups and fish. In a doxorubicin model, its supplementation was associated with the reduction of Na^+^/H^+^ exchanger-1, an OS-related membrane enzyme that participates in the pathogenesis of cardiovascular diseases [73].

### 5.15. Ginkgo biloba

Ginkgo biloba (GB) is an Asian plant with edible nuts and antioxidant properties. GB contains many bioactive components that positively regulate antioxidant enzyme expression and reduce ROS damage. Ginkgolide A (GA) is a component extracted from GB [74]. In this study, the therapeutic potential of GA was tested in rats with aortic constriction to protect against adverse CR. Ginkgolide demonstrated cardioprotection through the reduction of OS [75].

### 5.16. Roselle

Roselle is a tropical plant, scientifically named Hibiscus sabdariffa, consumed as tea, juice, jelly, or preservative. Several therapeutic effects have been described, particularly for the treatment of hypertension. Its antioxidative capacity has been described and attributed to being rich in polyphenols, especially anthocyanins. The experimental study in Table 2 describes the attenuation of cardiac hypertrophy in vivo and in vitro, with positive results for OS [76].

### 5.17. Atractylodis macrocephalae Rhizoma

Atractylodis macrocephalae rhizoma (AMR) is an edible Chinese medicinal herb with antioxidant activity attributed to phenolics (phenolic acids and flavonoids), responsible for its antioxidant effects through metal chelation and ROS scavenging [77]. In Table 2, the authors found that AMR prevents CR in a model induced by isoprenaline. The attenuation of OS was shown by reduced levels of MDA [78].

### 5.18. Herba Houttuynia

Herba houttuynia (HC) is an edible medicinal plant that grows in moist and shaded areas. They can be added to soups, salads, or meat and are used especially in Vietnamese cuisines. The antioxidant activities of HC were attributed to the presence of several polyphenols [79]. A study showed that reduced CR parameters evaluated in the acute phase of damage induced by acute hyperlipidemia were associated with beneficial OS variables [80].

**Table 1:** Diets, macro-, and micronutrients investigated regarding their potential therapeutic antioxidant effects on cardiac remodeling.

Investigated Compound	Model	Cardiac Remodeling Effect	Redox Effect	Reference
Hypocaloric diet	Rats (obesity) Rats (metabolic syndrome)	↓ hypertrophy ↓ hypertrophy ↓ fibrosis ↓ diastolic dysfunction	↓ HO-1, ↓NQO1 ↓ NOX	[8] [9]
Ketogenic diet	Rats (underlying)	↓ hypertrophy ↓ fibrosis	↑ SOD, ↑ GPx, ↑ CAT, ↓ MDA ↓ 3-NT	[10]
Sardine oil-loaded microparticle	Cardiomyoblasts (doxorubicin toxicity)	↓ apoptosis	↓ ROS	[12]
Omega-3 and lignan	Rats (aortic stenosis)	improved LV contractile dysfunction, ↓ apoptosis, ↓ fibrosis	↑ SOD, ↑ GPx, ↑ GST, ↑ MPO, ↓ MDA	[14]
Omega-3	Rats (doxorubicin toxicity)	↓ cardiac histopathological damages, ↓ apoptosis	↑ SOD, ↑ GPx, ↓ MDA	[15]
α-Linolenic acid	Rats (doxorubicin toxicity)	improved cardiac function, ↓ cardiac histopathological damages	↑ SOD, ↑ GPx, ↑ CAT, ↑ Nrf2 ↓ MDA	[16]
ATRA	Cardiomyocytes (mechanical stretch) Cardiomyocytes (doxorubicin) Rats (pressure overload) Rats (doxorubicin)	↓ apoptosis ↓ cells death ↓ hypertrophic, fibrosis, and apoptosis ↓ myocardial histoarchitecture damages	↓ ROS, ↑ SOD ↓ ROS, ↑ HO-1, ↑ SOD, ↑ Nrf2 ↑ SOD ↑SOD, ↑ GPx, ↑ CAT, ↑GSH, ↓ MDA	[19] [20] [21] [22]
Beta-carotene	Rats (cigarette smoking)	↓ hypertrophy, preserved fibers morphological aspects	↓ LH	[23]
Folic acid	Rats (monocrotaline) Rats (aging process) Rats (high-fat diet–obesity)	↓ myocyte proliferation ↓ hypertrophy, ↓ fibrosis, ↓ apoptosis preserved LV function ↓cardiac dilatation, ↓ systolic dysfunction, ↓fibrosis	↓ SOD ↓ MDA, ↓ 8-OHDG ↑ CAT, ↑ GSH, ↓MDA	[25] [26] [27]
Vitamin E	Rats (diabetic cardiomyopathy)	improved LV systolic and diastolic function	↓ 8-iso PGF_2α_, ↓ GSSG	[28]
Combination of vitamins	Rabbits (tachycardia induced) Rabbits (myocardial infarction)	attenuated the increase of EDD and a decrease of FS attenuated LV dilation and dysfunction	↑ GSH/GSSG ↓ 8-oxo-dG and ↑ GSH/GSSG ↓ 8-OHDG, ↓ 4-HNE	[29] [30]
Zinc	Rats (myocardial infarction) Rats (diabetic cardiomyopathy)	↓ systolic and diastolic disfunction ↓ systolic and diastolic disfunction And ↓ fibrosis	↑ CAT, ↓ SOD ↑ GSH ↑ MT	[32] [34]
Selenium	Rats (myocardial infarction)	↓ necrosis, ↓ dilation of LV	↑ GPx, ↑TrxR	[36]
Magnesium	Rats (L-NAME hypertension)	↓ hypertrophy, restoring contractile dysfunction	↓ O_2_^−^, ↓ H_2_O_2_, ↓ Carbonyl	[39]

ROS = reactive oxygen species, SOD = superoxide dismutase, HO-1 = heme-oxygenase-1, Nrf2 = nuclear factor erythroid 2-related factor 2, GPx = glutathione peroxidase, CAT = catalase, GSH = reduced glutathione, MDA = malondialdehyde, LH = lipid hydroperoxide, 8-OHDG = 8-hydroxy-2′-deoxyguanosine, 8-iso PGF_2α_ = 8-iso-prostaglandin F_2α,_ GSSG = oxidized glutathione, GSH/GSSG = reduced glutathione/oxidized glutathione, 8-oxo-dG = 8-oxo-7,8-dihidro-2′-desoxiguanosina, 4-HNE = 4-hydroxy-2-nonenal, MT = cardiac-specific metallothionein, TrxR = thioredoxin reductase, O_2_^−^ = superoxide anion, H_2_O_2_ = hydrogen peroxide, MPO = myeloperoxidase.

**Table 2:** Phytochemicals/phytonutrients investigated regarding their potential therapeutic antioxidant effects in cardiac remodeling.

Investigated Compound	Model	Cardiac Remodeling Effect	Redox Effect	Reference
Tomato	Rats (myocardial infarction)	improved diastolic dysfunction, ↓ interstitial fibrosis, ↓ hypertrophy	↓ LH	[45]
Spondias	Rats (tobacco smoke) Rats (myocardial infarction) Rats (ISP— cardiotoxicity)	↓ hypertrophy ↓hypertrophy, ↓ fibrosis ↓ hypertrophy, ↓ disruption and fragmentation myofibrils	↑ SOD, ↑ GPx, ↓ LH ↓ LH ↑ SOD, ↑ CAT ↓ MDA, ↑ GSH	[48] [49] [50]
Açaí	Rats (high-fat-diet) Rats (Ischemia-Reperfusion) Rats (doxorubicin cardiotoxicity)	↓ hypertrophy Poorer diastolic function Improved systolic function	↑ GPx ↑ CAT, ↑ Nrf2 ↑ CAT, ↑SOD, ↑ GPx, ↓ LH ↓ LH	[52] [53] [54]
Jaboticaba	Rabbits (doxorubicin cardiotoxicity) Rats (myocardial infarction)	↓ hypertrophy Improved diastolic function, ↓ fibrosis	↓ NT, ↓ MDA ↑ GPx, ↓ LH	[55] [57]
Bergamot	Rats (doxorubicin cardiotoxicity)	Improved systolic function ↓ hypertrophy, ↓ apoptosis	↓ ROS, ↓ MDA ↓ 8-OHDG	[58]
Orange juice	Rats (myocardial infarction) Rats (doxorubicin cardiotoxicity)	improved systolic and diastolic function, ↓ hypertrophy improved systolic and diastolic function, ↓hypertrophy	↓GPx, ↑ HO-1 ↑ SOD, ↑GPx, ↑ CAT ↑ LH	[59] [60]
Red raspberry	Rats (obese diabetic)	↓ levels of proteins associated involved in CR	↓ NQO1, ↓GSTA4, ↓GSTP1	[81]
Blueberry	Rats’ Cardiomyocytes (norepinephrine)	↓ hypertrophy, ↓cell death ↑ contractile function	↑ SOD, ↑ CAT	[62]
Cranberry	Rats (doxorubicin toxicity)	↓histopathological lesions	↑ GPx ↓ MPO, ↑ GSH, ↓ MDA	[63]
Cocoa–Carob blend	Rats (diabetic fatty)	mitigated cardiac dysfunction, hypertrophy, and fibrosis	↓ NOX, ↓ ROS ↑ SIRT1, ↑Nrf2	[65]
Rosemary	Rats (myocardial infarction)	Increased diastolic function ↓ hypertrophy	↓ SOD, ↓ LH	[66]
Camella sinensis	Humans (dialysis patients) Rats (doxorubicin cardiotoxicity Rats (myocardial infarction) Rats (doxorubicin cardiotoxicity)	↓ hypertrophy Preserved microarchitecture improved systolic and diastolic dysfunction, ↓ hypertrophy ↓ hypertrophy	↓ p22 phox, ↑ HO-1, ↓ fosforilaion ERK1/2 ↑ GPx, ↑ GSH, ↑ GST, ↑ SOD, ↑ CAT, ↓ MDA ↓ SOD, ↓ CAT, ↑ Nrf-2, ↓ Carbonyl ↓ LH ↑ SOD and GPx	[68] [82] [83] [84]
Moringa	Rats (myocardial infarction) Rats (isoproterenol cardiotoxicity) Rats (isoproterenol cardiotoxicity)	attenuating contraction dysfunction, ↓ infarction area, apoptosis, and fibrosis ↓ necrosis, inflammatory infiltrate, and preservation of the myofibrillar structure ↓ improved systolic and diastolic function, preserved microarchitecture, ↓ myonecrosis	↓gp91 phox ↑ SOD, ↑ CAT, ↑ GPx, ↑ GSH, ↓ MDA, ↓ ROS ↑ SOD, ↑ CAT, and GPx ↓ MDA	[85] [86] [71]
Citronellal	Rats (doxorubicin cardiotoxicity)	improvement of systolic function, ↓ apoptosis ↓ fibrosis	↑ SOD, ↑ GSH ↓ MDA ↓ NHE1	[73]
Ginkgolide A	Rats (aortic constriction)	↓ hypertrophy improved systolic cardiac function, ↓ apoptosis	↑ SOD, ↓ MDA, ↓NOX	[75]
Roselle	Rats (ISO myocardial infarction)	ameliorates cardiac diastolic dysfunction, ↓ hypertrophy, ↓ fibrosis	↑ SOD, ↑GSH, ↓NOX, ↓8-iso PGF_2α_	[76]
Atractylodis macrocephalae rhizoma	Rats (ISO myocardial infarction)	↓ hypertrophy, ↓ fibrosis	↓ MDA	[78]
Herba Houttuynia	Rats (hyperlipidemic induced)	↓ hypertrophy	↑ SOD, ↑ GST, ↑ GPx, ↑ Nrf2, ↑NQO1, ↑HO-1, ↓ carbonyl	[80]

LH = lipid hydroperoxide, SOD = superoxide dismutase, GPx = glutathione peroxidase, CAT = catalase, MDA = malondialdehyde, GSH = reduced glutathione, Nrf2 = nuclear factor erythroid 2–related factor 2, ROS = reactive oxygen species, 8-OHDG = 8-hydroxy-2′ –deoxyguanosine, HO-1 = heme-oxygenase-1, NQO1 = nicotinamide adenine dinucleotide phosphate dehydrogenase, quinone 1, GSTA4 = glutathione S-transferase alpha 4, GSTP1 = glutathione S-transferase P1, MPO = myeloperoxidase, NOX = nicotinamide adenine dinucleotide phosphate oxidase, SIRT1 = sirtuin-1, 3-NT = 3-nitrotyrosine, ERK1/2 = extracellular signal-regulated kinase-1/2, GST = glutathione S-transferase, TBARS = thiobarbituric acid reactive substances, NHE1 = Na^+^/H^+^ exchanger-1, 8-iso PGF_2α_ = 8-iso-prostaglandin F_2α._

## 6. Conclusions

CR process has been widely studied for at least three decades. The role of OS in the pathophysiology of cardiac remodeling has a strong scientific basis and several experimental studies support that antioxidant nutrients therapies can mitigate the damage caused by the OS. However, in clinical practice, translational medicine does not adequately support antioxidant therapy in the prevention of RC. Explanations for this are speculative.

Firstly, evaluating the experimental studies about antioxidant nutritional compounds, we realized that the comparison between them for summarizing their results is a challenge. Different OS induction models are used (ischemia, drugs), and even more distinct are the parameters for the evaluation of CR (several structural and/or functional parameters). Thus, if within the experimental studies themselves there is such a significant variety of models and results, the translation of what these findings may mean in clinical studies is even more uncertain.

The other problem can be in the propriety of nutrients offered. Some compounds, as vitamins, may have dose-dependent pro- and antioxidant properties, and it can sometimes be difficult to achieve dose equivalence used in vitro in human trials. Furthermore, we can consider that some compounds may require different cofactors for their antioxidant action, which may not be sufficiently available in humans. Another explanation refers to the duration of trials. Antioxidant therapy may be necessary for several years to reverse oxidative damage in humans and therefore their results may not be evidenced in short follow-ups [87].

Another possible explanation for the failure of clinical trials is that the wrong patients may have been studied. After all, there is a known difference between replacing a nutritional deficiency or supplementing. Perhaps only patients with specific deficiencies of these antioxidants nutrients show benefits in the administration of the same. In addition, can be necessary to screen patients with markers of OS and only those with a proven increase of these markers should be treated [87].

Finally, we highlight that drug options have already shown benefits in attenuating CR, such as angiotensin-converting-enzyme inhibitors and beta-blockers, for example. However, the evaluation of antioxidative nutrients in remodeling generally do separately from the already-established standard clinical treatment. Thus, we must evaluate the isolated results parsimoniously while acknowledging the lack of studies regarding the simultaneous use of nutrients and the already-established standard clinical treatment against CR [88].
